# Protective Effect of Standardized Extract of *Ginkgo biloba* against Cisplatin-Induced Nephrotoxicity

**DOI:** 10.1155/2013/846126

**Published:** 2013-11-25

**Authors:** Jie Song, Dan Liu, Liang Feng, Zhenhai Zhang, Xiaobin Jia, Wei Xiao

**Affiliations:** ^1^Key Laboratory of Delivery Systems of Chinese Materia Medica, Jiangsu Provincial Academy of Chinese Medicine, Nanjing, Jiangsu 210028, China; ^2^State Key Laboratory of New-Tech for Chinese Medicine Pharmaceutical Process, Jiangsu Kanion Pharmaceutical Co., Lianyungang, Jiangsu 222001, China

## Abstract

Cisplatin (CDDP) is a potent antitumor compound widely used with a notably side effect of nephrotoxicity inducing oxidative stress and apoptosis in kidneys. Standardized extract from the leaves of the *Ginkgo biloba* trees, labeled EGb761 (EGb), has been available on the market for its beneficial effects. The purpose of this study was to investigate the ability of EGb to prevent the nephrotoxic effect of CDDP and the mechanisms involved. Our results showed that EGb treatment restored the levels of creatinine, BUN, MDA, NO, SOD, CAT, GPx, and GSSG/GSH ratio in kidneys after CDDP injection. EGb also exhibited a tendency to decrease the elevated NF-**κ**B translocation and caspase-3 protein levels in CDDP-treated kidneys. We further used a porcine kidney proximal tubular epithelial (LLC-PK1) cell line, finding that EGb accordingly inhibited ROS accumulation and iNOS increase induced by CDDP *in vitro*. EGb also attenuated I**κ**B degradation and p65 NF-**κ**B phosphorylation triggered by CDDP in LLC-PK1 cells. But EGb failed to influence CDDP-stimulated caspase cascade. These findings suggested that EGb's renoprotective effect might be mediated by not only its well-known antioxidant activity but also the anti-inflammatory activity.

## 1. Introduction

Cisplatin (CDDP), a noncycle-dependent cytotoxic platinum derivative, has been frequently used in different solid tumors, including gastric, testicular, urologic, head, neck, and ovarian cancer [1]. Despite the therapeutic benefits, its use in clinical practice is often limited ascribing to dose-related toxicity. Clinical application shows that CDDP-induced nephrotoxicity can even force an interruption of the treatment of cancers. CDDP has multiple intracellular effects, including regulating genes, causing direct cytotoxicity with reactive oxygen species, and inducing apoptosis. The nephrotoxic potential of CDDP has been attributed to the overgeneration of reactive oxygen species (ROS) induced by the accumulation of CDDP in the renal tubular cells. Additionally, the activity reduction of antioxidant enzymes also causes morphological damage to the intracellular organelles [2]. Recent research has showed a significant new insight into the mechanisms leading to inflammation in CDDP-induced acute kidney injury [3]. There is a growing body of evidence indicating that CDDP induces remarkable activation of NF-*κ*B in kidneys, and the inhibition of NF-*κ*B activation is capable of attenuating CDDP-induced renal injury [4, 5]. 

*Ginkgo biloba* has been used in traditional Chinese medicine for thousand years. Clinically, it has been prescribed to treat Alzheimer's disease and cognitive deficits. Its purported biological effects include free radical scavenging, antiapoptotic, anti-inflammatory, and antioxidative activities [6]. EGb 761 (EGb) is a standardized, concentrated extract of *G. biloba* leaves and has been available on the market for cardiovascular disease. EGb was developed and put on market in the early 1970s by IPSEN in France and Dr. Willmar Schwabe Pharmaceuticals in Germany. EGb contains 24% flavonol glycosides (the flavonoid fraction) and 6% terpene lactones (terpenoid fraction). The flavonoid fraction is primarily composed of quercetin, kaempferol, and isorhamnetin. The terpenoid fraction primarily contains ginkgolides A, B, C, J, and M, as well as bilobalide. EGb shows a strong antioxidative property that directly scavenges ROS [7]. EGb also effectively alleviated CDDP-induced toxicity in reproductive system through decreasing p65 NF-*κ*B expression in testicular tissues [8]. Moreover, the vascular rescuing effects of EGb might be channeled through inhibiting inducible nitric oxide synthase (iNOS) and thereby nitric oxide (NO) formation. EGb also exerted neuroprotective and antiapoptotic effect on neuronal death and inhibited caspase-3 activity in cultured neurons which had been pretreated with hydroxyl radicals [9]. 

In our research, CDDP induced severe nephrotoxicity accompanied with malonaldehyde (MDA), NO, and ROS elevation. EGb could attenuate CDDP-induced nephrotoxicity via reducing oxidative stress markers and alleviating inflammation. MDA and NO increments were also diminished by EGb, which was consistent with previous research [10, 11]. However, the anti-inflammatory characteristic of EGb against nephrotoxicity has not been focused on. Herein, we further performed our research both *in vivo* and *in vitro* to explore the in-depth mechanism underlying the nephroprotective capability of EGb.

## 2. Materials and Methods

### 2.1. Reagents and Materials

Standardized EGb 761 is obtained from the pharmacological company IPSEN (Paris, France). CDDP, Tween 20, bovine serum albumin, and sodium dodecyl sulfate were purchased from Sigma Chemical Co. (St. Louis, MO, USA). The various antibodies were provided as follows: iNOS (Abcam, Cambridge, MA, USA), I*κ*B, p65 NF-*κ*B, phospho-p65 NF-*κ*B (Santa Cruz Biotechnology, Santa Cruz, CA, USA), and caspase-3, -9, and -8 (Cell Signaling Technology Inc., Danvers, MA, USA). All other chemicals were of analytical grade and were obtained commercially. 

### 2.2. Animals and Experimental Protocols

Female Sprague-Dawley rats (250–300 g) were obtained from the SLAC Lab Animal Center of Shanghai (Shanghai, China). Rats were maintained on a standard diet and water ad libitum. The experimental procedures were approved by the Animal Ethics Committee of Jiangsu Provincial Academy of Chinese Medicine. Rats were divided in the following groups: the control group (saline for 10 consecutive days through oral gavages); EGb group (200 mg/kg for 10 consecutive days through oral gavages); CDDP group (intraperitoneal injection once on the 5th day, 10 mg/kg); EGb + CDDP (EGb was administrated for 10 consecutive days through oral gavages and CDDP of 10 mg/kg was injected once on the 5th day) [5]. Blood and kidney tissues were collected on the 10th day after sacrificing the animals.

### 2.3. Histology

All kidney samples were processed and embedded in paraffin. Sections were cut at 5 *μ*m thicknesses on a rotary microtome. After stained with hematoxylin and eosin, the slides were observed and the photos were taken using binocular Olympus DX45 microscope. 

### 2.4. Biochemical Assay

Before sacrificed, the blood of rats was collected and centrifuged at 1411 g for 10 min to measure serum BUN and creatinine using autoanalyzer (Type 7020, Hitachi, Japan). The supernatant of tissue homogenates was used for the measurements of NO, MDA, glutathione oxidized form (GSSG), glutathione reductase (GSH), superoxide dismutase (SOD), catalase (CAT), and glutathione peroxidase (GPx) levels. MDA was determined spectrophotometrically at 535 nm [12]. NO levels were determined by the Griess reaction [13]. SOD and CAT activities were determined according to recently described methods [14]. GSSG and GSH levels were analyzed with a fluorescence microscope [15]. The measurement of GPx activity was performed based on monitoring of the oxidation of NADPH at 340 nm [16].

### 2.5. *In Situ* Detection of Apoptosis Using the TUNEL Assay

In order to evaluate the *in situ* apoptosis in kidney tubular cells, the terminal deoxynucleotidyl transferase-mediated nick end labeling (TUNEL) method was performed in the present study. Briefly, TUNEL staining was performed with the *In Situ* Cell Death Detection Kit from Keygen Biotech. Co., Ltd., Nanjing, China. A DAPI filter was used to detect DAPI staining (blue color), and an FITC filter was used to detect TUNEL staining (green color). TUNEL positive and DAPI positive staining patterns were photographed under a fluorescence microscopy (Zeiss Axio Observer A1). 

### 2.6. Immunohistochemical Studies

Sections (5 *μ*m) from paraformaldehyde-fixed tissues were dewaxed with xylene and graded ethanol series (100%, 95%, and 70%, v/v, 2 min each) and then washed in distilled water. The sections were incubated in 3% hydrogen peroxide to quench the activity of endogenous peroxidase. Sequentially, the slides were placed in citrate buffer and heated at 100°C to retrieve antigens. The slides were incubated with antiphospho-p65 NF-*κ*B antibody for 1 h, followed by incubation with horseradish peroxidase-conjugated secondary antibody. Sections were then washed with distilled water, incubated with diaminobenzidine-hydrogen peroxide, and counterstained with hematoxylin. Immunoreactivity was identified as brown nuclear in kidney sections counterstained with hematoxylin.

### 2.7. Cell Culture and Treatment

LLC-PK1 cells (porcine kidney proximal tubular epithelial cells) were purchased from American Type Culture Collection (Manassas, VA). Cultures were maintained in Medium 199 supplemented with 5% fetal bovine serum (FBS), 100 U/mL penicillin, and 100 *μ*g/mL streptomycin. Cells were maintained in a humidified incubator with 5% CO_2_ at 37°C. When reaching 80% confluent, LLC-PK1 cells were preincubated with EGb (200, 100, and 50 *μ*g/mL) for 24 h. CDDP of 50 *μ*M was used to induce apoptosis at 37°C for 12 h. At the end of the treatment, these cells were harvested and used for ROS detection and protein extraction. 

### 2.8. Western Blotting

Isolation of whole fractions of proteins from kidneys was conducted by homogenizing kidneys in RIPA buffer. The homogenate was centrifuged at 30520 g for 20 min and the supernatant was stored at −75°C until use. Nuclear protein extraction was carried out with the extraction kit (Keygen Biotech. Co., Ltd., Nanjing, China). Kidneys tissues or LLC-PK1 cells were homogenized in ice-cold buffer A and then centrifuged at 30520 g for 20 min. The pelleted nuclei were resuspended in buffer B. Lysates were then centrifuged at 30520 g for 10 min again. The obtained supernatants containing the nuclear proteins were collected and stored at −80°C.

Aliquots of the lysates were separated by 10% SDS-PAGE and transferred to polyvinylidene fluoride membranes. The membranes were blocked in TBS buffer containing nonfat milk for 1 h and then incubated with primary antibodies (iNOS, phospho-p65 NF-*κ*B, p65 NF-*κ*B, I*κ*B, and caspase-3, -8, and -9). The membranes were then washed and incubated with secondary antibodies for 1 h. Membranes were finally developed with the electrochemiluminescence detection reagents, and Image-Pro Plus (IPP) software for densitometry analysis is applied for the quantification of protein levels.

### 2.9. Reverse Transcriptase-Polymerase Chain Reaction (RT-PCR)

Total RNA was isolated from LLC-PK1 cells with TRIzol Reagent (Invitrogen, Carlsbad, CA). Integrity of isolated total RNA was examined by 1% agarose gel electrophoresis and RNA concentration was determined by UV-light absorbance at 260 nm. iNOS sense primer: 5-AGCTCCCCATTCTGAAGCCC-3; iNOS antisense primer: 5-TGGAGCACGCTGAACACCTC-3; GAPDH sense primer: 5-AAGGTCGGTGTGAACGGATTT-3; GAPDH antisense primer: 5-AGATGATGACCCTTTTGGCCC-3. RT-PCR analysis was performed as follows: DNA was denatured at 94°C for 3 min and cycled immediately for 35 cycles: denaturing at 94°C for 45 s, annealing at 56°C for 55 s, and extension at 72°C for 1 min. The PCR products were run on an agarose gel and stained with ethidium bromide and then photographed.

### 2.10. Measurement of Intracellular ROS Levels

For measuring the levels of intracellular ROS, LLC-PK1 cells after treatments were incubated with 50 *μ*M 2′,7′-dichlorodihydrofluorescein diacetate (H_2_DCFH-DA, Invitrogen, Carlsbad, CA) at 37°C in the dark for 30 min. Cells were then analyzed at an excitation wave length of 480 nm and an emission wave length of 525 nm by FACScan flow cytometer as previously described [17].

### 2.11. CDDP Excretion into the Urine

Urine sampling was scheduled according to the renal clearance method [18]. Urine samples were collected as follows: from 0 to 20 min and from 20 to 40 min before CDDP injection and from 0 to 20 min, from 20 to 40 min, from 40 to 60 min, from 60 to 80 min, and from 80 to 120 min after CDDP injection. In CDDP group, 10 mg/kg of CDDP was injected intraperitoneally to rats at time zero. After pretreated with EGb (200 mg/kg) for 5 consecutive days through oral gavage daily, rats received a intraperitoneal injection of CDDP at time zero. In CDDP + furosemide group, furosemide (20 mg/kg) solution was infused at a rate of 0.05 mL/min for 20 min before CDDP (10 mg/kg) was injected intraperitoneally. Constant infusion of furosemide was continued for an additional 20 min. The concentrations of unchanged CDDP in urine were determined by high-performance liquid chromatography (HPLC) using postcolumn derivatization as reported previously [19].

### 2.12. Statistical Analysis

Data are expressed as mean ± standard deviation (S.D.). The intergroup variation between various groups was measured by one-way analysis of variance (ANOVA) followed by Dunnett's multiple comparison test, and the comparisons between two groups were conducted by unpaired Student's *t*-test. Results were considered statistically significant when *P* < 0.05.

## 3. Results

### 3.1. Effect of EGb on CDDP-Induced Renal Dysfunction

To investigate the effects of EGb on CDDP-induced renal injury, rats were treated with CDDP, EGb, or CDDP plus EGb. Main components in EGb are listed in Table 1. The levels of traditional indicators of kidney damage, such as BUN and creatinine, were measured. CDDP treatment significantly increased the levels of BUN and serum creatinine compared to the control group (*P* < 0.05) as shown in Table 2. Administration of CDDP-treated rats with EGb (200 mg/kg) noticeably alleviated the elevated levels of BUN and serum creatinine from 84.21 ± 13.45 to 43.26 ± 9.53 mg/dL (*P* < 0.05) and from 1.72 ± 0.28 to 1.15 ± 0.23 mg/dL (*P* < 0.05), respectively. EGb alone did not exhibit any effect on BUN and creatinine levels. 

### 3.2. Effect of EGb on Renal Histology after CDDP Treatment

**Figure 1:**
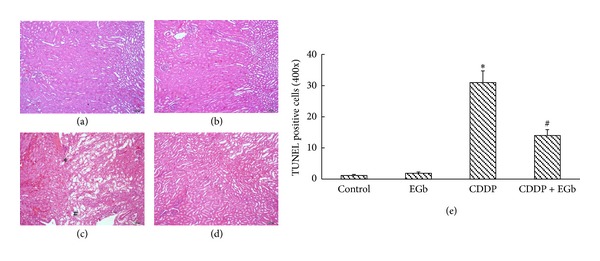
EGb treatment attenuated the kidney histological changes induced by CDDP in rats: (a) control rats; (b) EGb-only rats (200 mg/kg); (c) CDDP-treated rats (10 mg/kg); ^#^indicates necrotic debris, and *indicates proteinaceous effusion; (d) rats treated with EGb plus CDDP; (e) tubular damage was graded using the percentage of cortical tubules showing epithelial necrosis: 0 = normal; 1 ≤ 10%; 2 = 10–25%; 3 = 26–75%; 4 ≥ 75%. Data represent mean ± S.D. from three independent experiments. *P < 0.05 versus saline; ^#^ *P* < 0.05 versus CDDP. Original magnification, ×100.

The experiment was conducted to evaluate whether EGb can ameliorate CDDP-induced renal tubular damage. As presented in Figure 1, proteinaceous effusion, sloughing of proximal tubular epithelium, tubular vacuolar degeneration, and necrosis was evident in the CDDP group. As expected, less histological damage was observed in renal tubules in the CDDP plus EGb group. EGb dramatically declined the tubular necrosis scores from 2.65 ± 0.24 to 0.96 ± 0.31 after CDDP treatment.

### 3.3. Effect of EGb on Biochemical Markers of Oxidative Stress *In Vivo*

Oxidative stress has been regarded as one of the underlying mechanisms of CDDP-induced acute kidney injury [3]. Since EGb exhibited potent superoxide scavenging effect [20], we evaluated whether EGb (200 mg/kg) could modulate CDDP-induced renal oxidative stress by measuring lipid peroxidation level and activity of enzymes involved in antioxidation. Results in Table 3 showed that CDDP significantly increased the level of MDA and NO in the kidney tissues, compared to the control. Coadministration of EGb plus CDDP generated a significant decrease in MDA and NO levels. As shown in Table 2, GSSG/GSH ratio was enhanced as expected in CDDP-treated rats, whereas EGb attenuated the increment of GSSG/GSH ratio. The renal levels of SOD, CAT, and GPx were considerably reduced after CDDP treatment. EGb effectively diminished CDDP-induced depletion of SOD, CAT, and GPx levels. 

### 3.4. Effects of EGb on NF-*κ*B Activation and Translocation into Nucleus *In Vivo*

**Figure 2:**
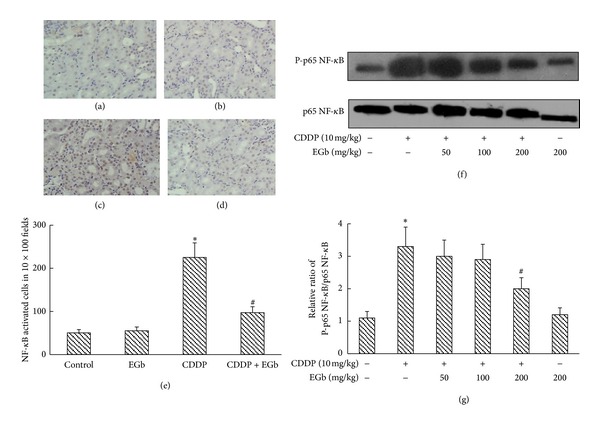
EGb inhibited NF-*κ*B activation in CDDP-induced acute renal injury in rats. ((a)–(e)) Immunohistochemical detection of translocational phospho-p65 NF-*κ*B in renal tissues. (a) Control, (b) EGb, (c) CDDP, and (d) CDDP + EGb. In EGb + CDDP group, nuclear stainings (grown) in renal tubular cells were significantly reduced compared with CDDP treated group. Magnification, ×100. (e) The number of NF-*κ*B activated cells was summarized. (f) Phospho-p65 and p65 expressions in nuclear and cytosolic protein extracts from CDDP-injected rats were analyzed by western blotting. Data represent mean ± S.D. from three independent experiments. *P < 0.05 versus saline; ^#^ *P* < 0.05 versus CDDP.

NF-*κ*B plays a key role in the inflammation process during nephrotoxicity. We determined NF-*κ*B activation by measuring the activation of the p65 subunit in western blotting analysis. As parallel with p65 protein expression, the phosphorylation of nuclear p65 was increased by the treatment of CDDP. However, this increase was significantly downregulated by the treatment of EGb (200 mg/kg) (Figure 2). In the following immunohistochemical studies, greater and intensive translocated p65 staining in the nucleus of the cells was seen in the renal cortices and outer medullae of CDDP-treated rats than the control group and EGb-only group (200 mg/kg). Immunohistochemistry assay confirmed the result obtained from the western blot that EGb treatment blunted CDDP-induced activation and translocation of p65. Correspondingly, the number of NF-*κ*B activated cells was reduced from 225 ± 34 to 97 ± 14 in 10 × 100 fields.

### 3.5. Effects of EGb on CDDP-Induced Apoptosis *In Vivo*

**Figure 3:**
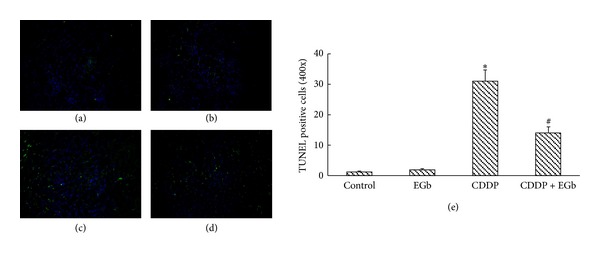
EGb inhibited the tubular apoptosis in rats induced by CDDP. TUNEL assay was performed from rats kidney: (a) control rats; (b) EGb-only rats (200 mg/kg); (c) CDDP-treated rats (10 mg/kg); (d) rats treated with EGb plus CDDP. As described in the methods, fragmented DNA labelling corresponds to green spots. (e) Quantitative analysis of TUNEL staining. Data represent mean ± S.D. from three independent experiments. *P < 0.05 versus saline; ^#^ *P* < 0.05 versus CDDP. Original magnification, ×100.

Renal tubular apoptosis has been suggested as a mechanism on CDDP-induced acute kidney injury [3]. We further determined EGb's ability to reduce CDDP-mediated apoptosis in the kidney by TUNEL assays. Increased TUNEL staining was observed in CDDP-treated rats on the 5th days after CDDP injection (Figure 3). Treatment with EGb (200 mg/kg) after CDDP injection substantially reduced the intensity and distribution of TUNEL staining, which implied that the apoptotic cascade might play a key role in EGb's renoprotective effect. Accordingly, TUNEL positive cell numbers were reduced from 31 ± 3.7 to 14 ± 1.9 in 400 × fields (*P* < 0.05). No significant TUNEL positive staining was observed in the EGb-only group.

**Figure 4:**
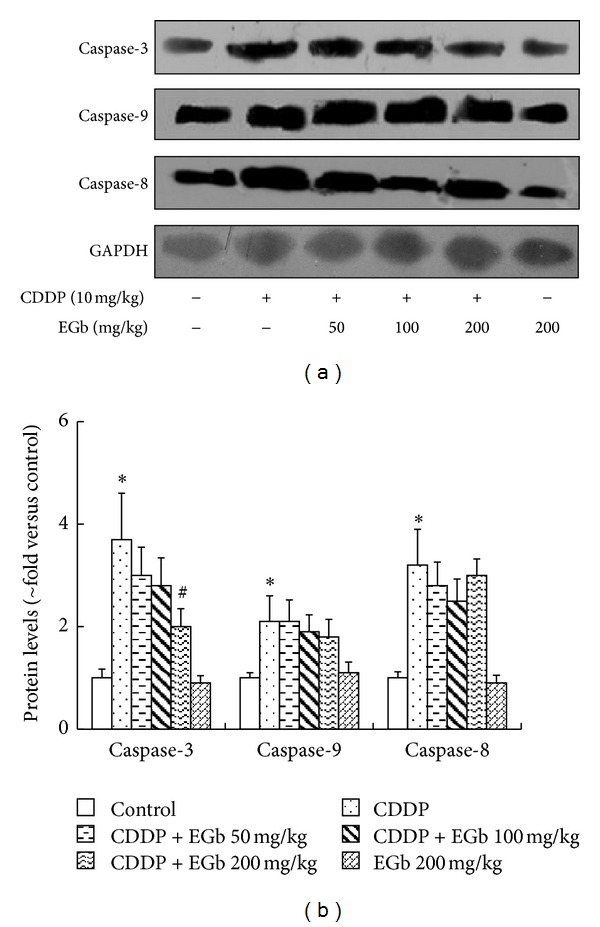
EGb reduced the caspase-3 activity in CDDP-injected rats. Rats were treated with or without EGb (200, 100, and 50 mg/kg) plus CDDP (10 mg/kg). Caspase-3, caspase-9, and caspase-8 protein levels were analyzed by western blotting. Protein expressions were semiquantified by densitometry analysis. Data represent mean ± S.D. from three independent experiments. *P < 0.05 versus saline; ^#^ *P* < 0.05 versus CDDP.

We further investigated the protein expressions of caspases of rat kidneys in the EGb plus CDDP group. As expected, western blotting analysis showed that apoptotic caspase-3, -8, and -9 protein levels were significantly higher in the CDDP-injected rats than the control group and EGb-only group (Figure 4). After CDDP administration, EGb (200 mg/kg) significantly reversed the increased caspase-3 protein expression in kidneys, but caspase-8 and -9 expressions were unchanged.

### 3.6. Effects of EGb on LLC-PK1 Cells after CDDP Treatment *In Vitro*

**Figure 5:**
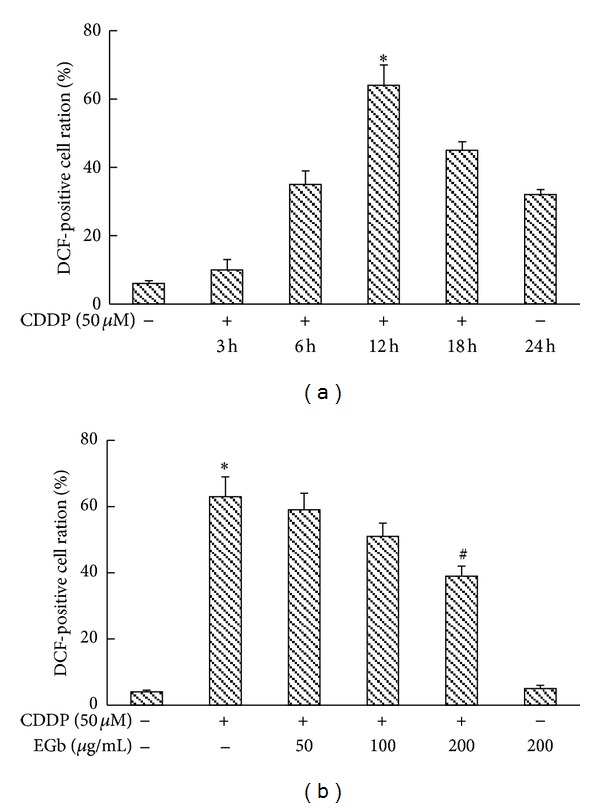
EGb repressed CDDP-stimulated generation of ROS in LLC-PK1 cells. (a) LLC-PK1 cells were incubated with 50 *μ*M CDDP for the indicated time periods. (b) LLC-PK1 cells were preincubated with EGb (200, 100, and 50 *μ*g/mL) for 24 h and then incubated with 50 *μ*M CDDP at 37°C for 12 h. The generation of ROS was measured by using the fluorescent dye DCF-DA in FACScan flow cytometry. The corresponding linear diagram of FACScan flow cytometry is shown. Data represent mean ± S.D. from three independent experiments. *P < 0.05 versus saline; ^#^ *P* < 0.05 versus CDDP.

*In vivo* results revealed that oxidative stress was closely related to the effect of EGb on CDDP-injected rats. To verify our hypothesis that EGb treatment could mitigate CDDP-induced nephrotoxicity via inhibiting ROS, we then determined ROS accumulation in LLC-PK1 cells. As shown in Figure 5, we observed that depending on the concentration, CDDP (50 *μ*M) could rapidly induce ROS production at 6 h afterinduction, and ROS level was further augmented over the next 18 h of the study. The ratio of DCF-positive cells drastically decreased in LLC-PK1 cells preincubated with EGb of 200 *μ*g/mL for 24 h (*P* < 0.05).

**Figure 6:**
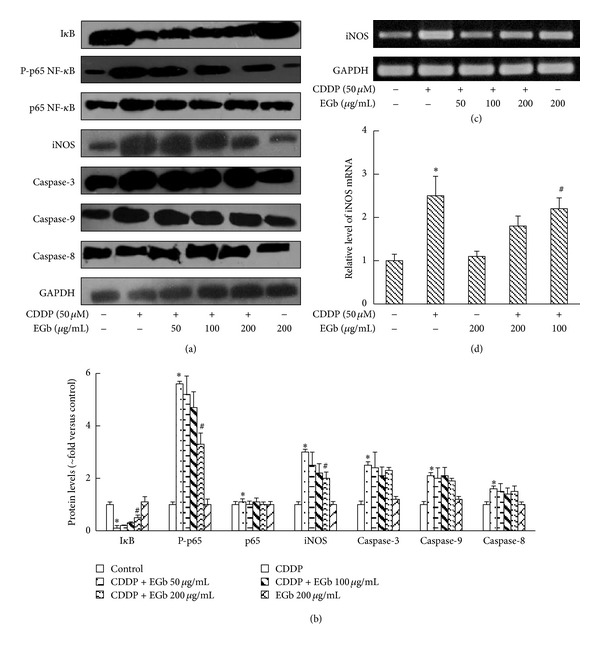
EGb suppressed CDDP-induced I*κ*B degradation, p65 phosphorylation, and iNOS expression in LLC-PK1 cells. (a) LLC-PK1 cells were preincubated with EGb (200, 100, and 50 *μ*g/mL) for 24 h and then incubated with 50 *μ*M CDDP at 37°C for 12 h. I*κ*B, iNOS, phospho-p65, p65, caspase-3, caspase-9, and caspase-8 protein levels from LLC-PK1 cells extracts were analyzed by western blotting. (b) Protein expressions were semiquantified by densitometry analysis. (c) iNOS mRNA levels were analyzed with RT-PCR and then semiquantified. Data represent mean ± S.D. from three independent experiments. *P < 0.05 versus saline; ^#^ *P* < 0.05 versus CDDP.

As noted earlier, degradation of I*κ*B allows the nuclear localization of NF-*κ*B and subsequent transcriptional activation of target genes. We then treated cultured LLC-PK1 cells with 50 *μ*M CDDP or CDDP plus EGb (200, 100, and 50 *μ*g/mL) to determine whether I*κ*B is involved in the process. As shown in Figure 6(a), EGb inhibited the degradation of I*κ*B as well as the phosphorylation of p65 NF-*κ*B induced by CDDP. However, EGb failed to exhibit any effect on the caspase cascade in LLC-PK1 cells, which was in consistence with the *in vivo* results. 

EGb was able to diminish the elevation of NO in CDDP-treated rats, and iNOS is responsible for NO abundance under oxidative stress [21, 22]. We further analyzed iNOS mRNA and protein level by the reverse transcription RT-PCR and western blotting method. Both protein and mRNA levels of iNOS increased significantly in LLC-PK1 cells, and EGb effectively reversed the upregulation induced by CDDP (Figure 6).

### 3.7. Effect of EGb on the Cumulative Excretion of CDDP in Urine

**Figure 7:**
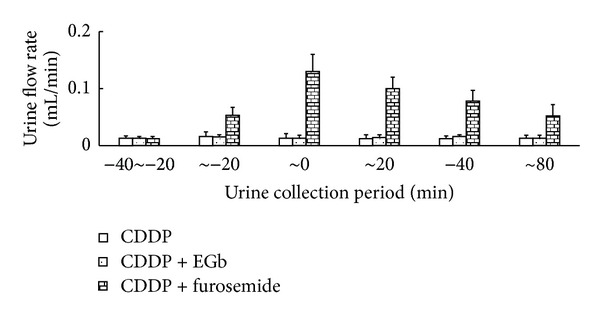
Effect of EGb and furosemide on urine output. In CDDP group, 10 mg/kg of CDDP was injected intraperitoneally to rats at time zero. CDDP + EGb group, EGb for 5 consecutive days through oral gavages and intraperitoneal injection of CDDP once on the 5th day. In CDDP + furosemide group, furosemide solution was infused at a rate of 0.05 mL/min for 20 min before CDDP was injected intraperitoneally. Constant infusion of furosemide was continued for an additional 20 min. Values are mean urine output ± S.D. in each urine collection period.

**Figure 8:**
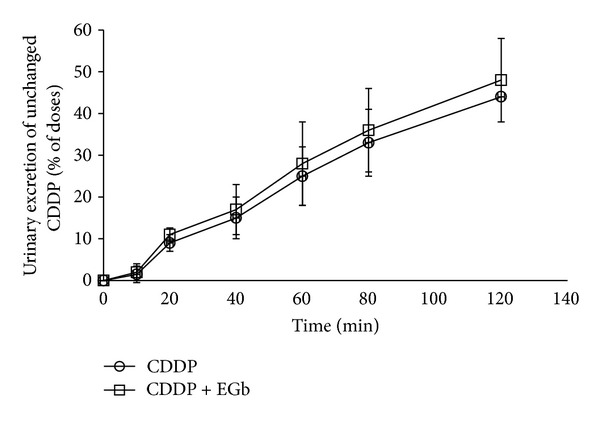
Effect of EGb on the cumulative excretion of unchanged CDDP in urine (percent of dose). In CDDP group, CDDP was injected intraperitoneally to rats at time zero. CDDP + EGb group, EGb for 5 consecutive days through oral gavages and intraperitoneal injection of CDDP once on the 5th day at time zero. Values are expressed as mean ± S.D.

It is well known that extensive diuresis achieved through hydration and administration of a diuretic (mannitol or furosemide) is effective for amelioration of CDDP nephrotoxicity [23]. Some herbs containing quercetin, kaempferol, and isorhamnetin, which are the main components in EGb, have been reported to have diuretic properties [24–26]. Herein, we investigated whether EGb had diuretic effect and explored the influence on CDDP excretion into the urine. The time courses of the urine flow rate were shown in Figure 7, and the maximum levels of urine output were about ~15-fold (furosemide) compared to CDDP alone and CDDP + EGb groups. There was no significant difference in urine flow rate between CDDP group and CDDP + EGb group. Likewise, EGb failed to modulate the cumulative excretion of CDDP (Figure 8).

## 4. Discussion

It is noteworthy that the therapeutic benefits of EGb rely on a combination of its constituents, each with its own pharmacological activity. Thus, it is not surprising that its therapeutic benefits are polyvalent or multifactorial. In this study, we demonstrated that EGb could restore renal injury, apoptosis, and inflammation induced by CDDP treatment and confirmed the important role of EGb's antioxidant and anti-inflammatory properties against CDDP-induced nephrotoxicity, in particular, via regulating iNOS expression and p65 NF-*κ*B translocation. 

In the past, studies on the pathogenesis of CDDP nephrotoxicity mainly focused on the direct toxicity such as oxidative stress [27]. The administration of antioxidants has been shown to ameliorate CDDP-induced toxicity in rats [28]. CDDP can cause an increase in lipid peroxide levels and a decrease in the activity of antioxidant defense enzymes that protect from lipid peroxidation in kidneys. This could explain the potent ability of EGb to diminish MDA level against CDDP treatment. Consistently, Inselmann et al. [29] reported that *Ginkgo biloba* extract could decrease CDDP-induced lipid peroxidation in rat renal cortical slices. Moreover, the decline of SOD and CAT activities as well as increment of GSSG/GSH ratio were exhibited after CDDP administration, resulting in the reduced ability of the kidney to scavenge toxic H_2_O_2_, O_2_^−^, and lipid peroxides. In our study, EGb was able to restore SOD, CAT, and GPx levels in kidney tissues. Lin and Chang [30] reported similar elevation in SOD and CAT activities in the epidermis and in the liver of rats after EGb treatment. In addition, the depletion of GSH redox cycle seems to be an essential factor that permits lipid peroxidation and ROS accumulation in the CDDP-treated rats [28]. GPx metabolizes H_2_O_2_ to water by using GSH as a hydrogen donor, resulting in the generation of GSSG. EGb was able to decrease GSSG/GSH ratio, probably by reversing the depletion of GSH concentration. 

CDDP initially triggers oxidative stress in the kidney, which is followed by a secondary wave of ROS/RNS (reactive nitrogen species) generation and an intense inflammatory response. ROS directly acts on cell components, including lipids, proteins, and DNA and destroys their structure. We demonstrated that EGb could reverse ROS accumulation in LLC-PK1 cells. RNS are originated from NO, which promotes oxidative stress-induced cell injury by formation of peroxynitrite anion (ONOO^−^), a potent prooxidant and cytotoxic intermediate [31]. NO can also enhance cellular injury by decreasing intracellular GSH levels [32]. NO is synthesized by a family of nitric oxide synthase (NOS) composed of three isoforms: neuronal NOS (nNOS), iNOS, and endothelial NOS (eNOS) [33]. Chirino et al. [34] reported that selective iNOS inhibition could reduce the CDDP-induced nephrotoxicity and nitrosative stress. Moreover, EGb can decrease NO production by inhibiting both gene expression and enzymatic activity of iNOS [10]. In our research, NO and iNOS levels surged following CDDP administration. EGb also inhibited inflammatory iNOS expressions in LLC-PL1 cells, the classic target of anti-inflammatory pathways. However, Gulec et al. [35]  previously reported that EGb protected against CDDP-induced nephrotoxicity by suppressing adenosine deaminase activities, and EGb showed a tendency to inhibit NO levels but with no statistical significance. Although not all the results are uniform, an effect on iNOS by EGb seems reasonable and accurate. 

The central role of cytokine activity and inflammation in CDDP nephrotoxicity has been focused on recently [36]. Among them, NF-*κ*B has been reported to coordinate the activation of a large network of chemokines and cytokines in the kidney following CDDP injection. At the same time, NF-*κ*B could be an important molecule that possibly couples ROS to regulate CDDP nephrotoxicity, further exacerbating renal tissue damage [37]. ROS can also activate NF-*κ*B that leads to increased expression of proinflammatory mediators which could intensify the cytotoxic effects of CDDP [38]. Moreover, antioxidants can attenuate ROS generation and NF-*κ*B activation and thus protect the renal cells against CDDP injury [39]. NF-*κ*B transduces oxidative stimuli to nucleus to modulate the expression of many genes involved in inflammatory responses, and one such gene is for iNOS [40]. Preventing NF-*κ*B and thus iNOS with antioxidants is effective in diminishing the CDDP-induced injury [41]. In our study, EGb reduced NF-*κ*B translocation both *in vivo* and *in vitro*, probably by stabilizing the upstream activator I*κ*B. 

In this study, EGb only caused a modest reduction in caspase-3 expression *in vivo.* This might reflect the incomplete suppression of caspase cascade by EGb. Accordingly, Luo et al. [42] revealed that EGb protected against neuron cell death with only a minor effect on caspase-3 expression. ROS production can be boosted by inducing mitochondrial dysfunction and caspase cascade activation via the disrupted respiratory chain or by depleting and inactivating glutathione and thus shifting the cellular redox status. Herein, the lack of any effect on caspase-8 and -9 expressions both *in vitro* and *in vivo* in our research suggested that caspase cascade was not a major pathway for EGb's protection against CDDP injury.

## 5. Conclusions 

In this study, we demonstrated that the anti-inflammatory property was involved in the renoprotective effect of EGb against CDDP-induced nephrotoxicity in SD rats and LLC-PK1 cells. In addition, we suggested that iNOS and NF-*κ*B were key mediators during the anti-inflammatory process, providing novel insights into the mechanism.

## Figures and Tables

**Table 1:** Contents of quercetin, kaempferol, and isorhamnetin in EGb.

Component	Contents (mg)^a^
Quercetin	12.1787
Kaempferol	11.7242
Isorhamnetin	3.5443

^a^Content means the quality of individual component in the *Ginkgo biloba* extracts per 100 mg.

**Table 2:** The effect of EGb on changes of tissue BUN, creatinine, and MDA as well as NO levels in control and study groups.

	Dose (mg/kg)	BUN (mg/dL)	Creatinine (mg/dL)	MDA (nmol/g wet tissue)	NO (*μ*mol/g wet tissue)
Control		11.12 ± 3.22	0.56 ± 0.12	6.55 ± 1.22	0.63 ± 0.12
CDDP	10	84.21 ± 13.45*	1.72 ± 0.28*	12.31 ± 3.27*	1.26 ± 0.39*
EGb	200	10.31 ± 2.63	0.58 ± 0.17	5.84 ± 1.18	0.72 ± 0.19
CDDP + EGb		43.26 ± 9.53^#^	1.15 ± 0.23^#^	8.03 ± 2.34^#^	0.89 ± 0.15^#^

Each value is expressed as mean ± S.D. (*n* = 10/group); *P < 0.05 versus saline; ^#^ *P* < 0.05 versus CDDP.

**Table 3:** The effect of EGb on changes in activities of antioxidant enzymes in control and study groups.

	Dose (mg/kg)	SOD (U/mg protein)	CAT (U/mg protein)	GPx (mU/mg protein)	GSSG/GSH ratio
Control		45.86 ± 6.24	13.23 ± 3.20	923.19 ± 176.32	0.65 ± 0.13
CDDP	10	19.13 ± 6.15*	6.25 ± 1.26*	667.23 ± 124.77*	1.16 ± 0.25*
EGb	200	47.71 ± 15.36	13.63 ± 2.16	925.46 ± 112.79	0.67 ± 0.17
CDDP + EGb		36.62 ± 12.25^#^	11.15 ± 1.29^#^	858.39 ± 198.63^#^	0.87 ± 0.19^#^

Each value is expressed as mean ± S.D. (*n* = 10/group); *P < 0.05 versus saline; ^#^ *P* < 0.05 versus CDDP.

## Abbreviations

Cisp: Cisplatin
EGb: EGb761
ROS: Reactive oxygen species
BUN: Blood urea nitrogen
MDA: Malonaldehyde
NO: Nitric oxide
SOD: Superoxide dismutase
CAT: Catalase
GPx: Glutathione peroxidase
GSSG: Glutathione oxidized form
GSH: Glutathione reductase
NF-*κ*B: Nuclear factor *κ*B
LLC-PK1 cells: Porcine kidney proximal tubular epithelial cells
I*κ*B: Inhibitor of NF-*κ*B
iNOS: Inducible nitric oxide synthase
RT-PCR: Reverse transcriptase-polymerase chain reaction
H_2_O_2_: Hydrogen peroxide
O_2_^−^: Superoxide anion
TUNEL: Terminal deoxynucleotidyl transferase-mediated nick end labeling
DAPI: 4′,6-Diamidino-2-phenylindole
HPLC: High performance liquid chromatography.
